# Osthole Alleviates D-Galactose-Induced Liver Injury *In Vivo* via the TLR4/MAPK/NF-κB Pathways

**DOI:** 10.3390/molecules28010443

**Published:** 2023-01-03

**Authors:** Zhe Ma, Lin Peng, Wenhui Chu, Pan Wang, Yongqian Fu

**Affiliations:** 1 School of Life Science, Taizhou University, Taizhou 318000, China; 2 Taizhou Key Laboratory of Biomass Functional Materials Development and Application, Taizhou University, Taizhou 318000, China; 3 Traditional Chinese Medicine Industry Development and Promotion Center of Pan’an County, Pan’an 322300, China

**Keywords:** osthole, aging, liver injury, inflammation, oxidative stress

## Abstract

Osthole, a coumarin derivative, is found in several medicinal herbs. However, the protective effects of osthole against D-galactose (D-Gal)-induced liver injury still remain unclear. In this study, osthole treatment effectively reversed D-Gal-induced liver injury, according to the results of liver HE staining, and improved ALT and AST activities. Feeding with D-Gal significantly increased MDA content, and reduced the level or activity of SOD, CAT and GSH-Px, which were all alleviated by osthole intervention. Meanwhile, osthole treatment significantly inhibited the D-Gal-induced secretion of pro-inflammatory cytokines, such as TNF-α, IL-1β and IL-6, in both serum and liver tissue. Investigations revealed that osthole ameliorated the D-Gal-induced activation of TLR4, MYD88 and its downstream signaling pathways of MAPK (p38 and JNK) and NF-κB (nucleus p65). Therefore, osthole mediates a protective effect against D-Gal-induced liver injury via the TLR4/MAPK/NF-κB pathways, and this coumarin derivative could be developed as a candidate bioactive component for functional food.

## 1. Introduction

Coumarins with 2H-benzopyran-2-one core structure are widely distributed in higher plants (such as the Umbelliferae and Rutaceae families), as well as various fruits, including carrots, cherries, citrus fruits, apricots, celery, parsnips and strawberries. Based on chemical structure, coumarins are mainly divided into six types, including simple coumarins, furanocoumarins, dihydrofuranocoumarins, phenylcoumarins, pyranocoumarins, and biscoumarins [1]. According to previous reports, coumarins exhibited anti-inflammatory, antibacterial, antiviral, antioxidant, antithrombotic, anti-Alzheimer’s disease (AD), antidiabetic, anticonvulsant, and antitumor effects, as well as the inhibition of lipoxygenase and the inhibition of xanthine oxidase [2]. Some identified chemicals belonging to coumarins possess attractive features, such as low molecular weight, simple structure, low side effects and minimal drug resistance, and these features make them suitable for development as drug candidates [3]. Moreover, coumarins have been used as additives in food and cosmetics due to their fungicide and antioxidant activities [4].

As a coumarin derivative, osthole (7-methoxy-8-(3-methyl-2-butenyl) coumarin) was first obtained from *Cnidium monnieri* (L.) Cusson, which is a traditional Chinese herb for treating male sexual dysfunction and other symptoms. According to the Pharmacopoeias of China and other countries, osthole content is listed as a key factor of quality of herbs such as *Fructus Cnidii* and *Angelicae pubescentis* radix. In previous study, osthole exhibited various pharmacological and biological effects, including antitumor, anti-inflammatory, neuroprotective, osteogenic, cardiovascular protective, antimicrobial and antiparasitic activities [5]. The antioxidant and anti-inflammatory activities of osthole have been well-acknowledged in several *in vitro* and *in vivo* models. In some *in vitro* studies, osthole scavenged ROS (e.g., hydroxyl radical, superoxide anion radical) [6] and exhibited anti-inflammation activity in various cell lines, including RAW 264.7 macrophage [7], SH-SY5Y cells [8], SW982 cells [9] and BV2 mouse microglia [10]. In these *in vitro* studies, osthole remarkably inhibited the expression of pro-inflammatory cytokines, such as TNF-α, IL-1β and IL-6. Meanwhile, the anti-inflammatory activity of osthole is also confirmed in animal models. Osthole was reported to effectively protect against ulcerative colitis by inhibiting the expression of TNF-α and reducing MPO activity in the colon [7]. In acute lung injury, osthole effectively reduced the expression of IL-6, IL-1β and TNF-α in serum and lung tissue, and pathological changes in the lung tissue significantly improved [11]. In renal ischemia–reperfusion-caused acute kidney injury, the administration of osthole effectively reduced the levels of TNF-α, IL-8 and IL-6 in serum and kidney tissue, and improved the pathological changes [12]. The protective role of osthole is proven to be associate with inflammation-related signal pathways, such as JAK/STAT, MAPK and NF-κB [5].

Aging is a gradual and biological process, and liver injury is characterized by steatosis, necrosis, the decreased regeneration of liver cells and finally liver failure. Currently, aging is attracting great attention in society because it is associated with various diseases, including diabetes, cancer, cardiovascular diseases, and Alzheimer’s disease [13]. Both inflammation and oxidative stress could be attributed to aging [14]. In D-Gal-induced liver injury mouse models, previous studies showed an increase in biochemical indicator levels (such as ALT, AST and ALP) in serum, and the oxidative stress index (such as SOD, MDA and GSH-Px) in liver [15,16]. Moreover, the increase in pro-inflammatory cytokines in the serum and liver, and the pathological changes in liver tissue confirm the relationship between inflammation and liver injury [17]. Some low molecular chemicals from natural resources are being developed as drug candidates with the ability of protecting against liver injury. The underlying mechanisms of these chemicals are related to inflammation. Torularhodin, from the aerobic yeast *Sporidiobolus pararoseus,* ameliorates oxidative stress and liver injury *in vivo* through the Nrf2/HO-1 signaling pathway, which is also known to be associated with inflammation [15]. The hepatoprotective effect of pyrroloquinoline quinone against alcoholic liver injury was related to the Nrf2-mediated NF-κB pathway [18]. These findings indicated that natural compounds with anti-inflammatory activity could be suitable targets, with hepatoprotective activity against alcoholic liver injury.

Currently, there is no obvious clue for the direct relationship between osthole and D-gal-induced liver injury. Depending on the protective effect of osthole on renal [12] and lung injury [11], and its anti-inflammatory activity, we hypothesized that this coumarin derivative may possess a hepaprotective effect on liver injury. Therefore, we analyzed the effects of osthole on an *in vivo* rat model of D-gal-induced liver injury to elucidate the underlying molecular mechanisms.

## 2. Results

### 2.1. Effect of Osthole on Body Weight and Liver Index

After sacrificing mice, the body weights (BW) and liver indexes of six groups, which were control, model, VE (vitamin E, positive group), and low-/medium-/high-dose osthole groups, were detected, as shown in Supplementary Materials Figure S1. In the model group, mice were intraperitoneally administered 200 mg/kg/BW D-galactose for 8 weeks to induce the liver injury. During the 8-week feeding period, BW in the model group was significantly reduced, compared to the control group (*p* < 0.01). BWs in the VE and experimental groups were significantly lower than in the control group (*p* < 0.01). However, there was no obvious difference between the VE (vitamin E, positive group) and experimental groups. The results in Figure S1 show a similar trend for liver indexes. In a previous study, it is reported that feeding with D-Gal triggers a remarkable decrease in BW and organ indexes [19]. The results in this study are accordance with this previous study, and also showed the protective effect of osthole against D-Gal-induced liver injury.

### 2.2. Effect of Osthole on Liver Pathological Changes

**Figure 1:**
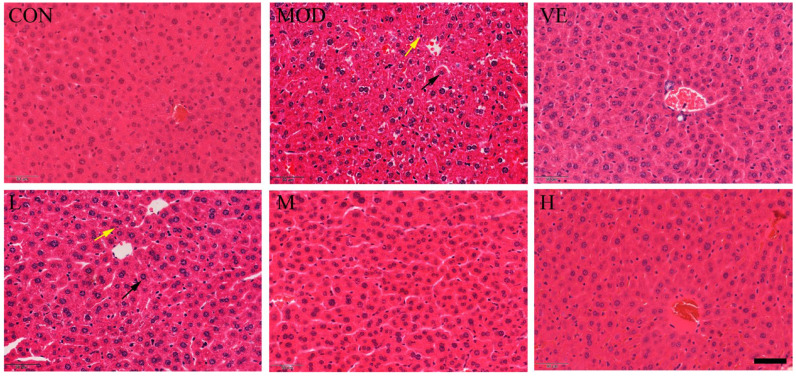
Effect of osthole on liver histology in D-Gal-injected mice, revealed by hematoxylin–eosin (HE) staining. CON, control group; MOD, model group; VE, positive group; L, D-Gal + 100 mg/kg osthole; M, D-Gal + 200 mg/kg osthole; H, D-Gal + 400 mg/kg osthole. Yellow and black arrows indicate inflammatory cells and enlarged hepatocytes, respectively. Scale bar: 50 µm and magnification power × 1000.

Compared to the healthy control group, the liver tissue of mice in the model feeding with D-Gal exhibited apparent inflammatory cell infiltration and enlarged hepatocytes around the central vein (Figure 1). After feeding with osthole, a disordered arrangement still existed, but the edema and inflammatory cell infiltration were significantly reduced, compared to the model group. Moreover, osthole significantly reduced the hepatic lobule structure, and liver tissue damage returned to a normal state in a dose-dependent manner. This result indicated that osthole can alleviate D-Gal-induced liver cell apoptosis and tissue damage.

### 2.3. Effect of Osthole on Biochemical Indicators and Pro-Inflammatory Cytokines in Serum

**Figure 2:**
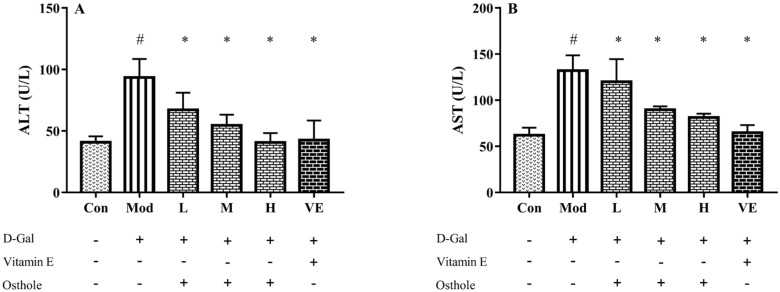
Effects of osthole on serum activity or levels of (**A**) ALT and (**B**) ASP in mouse D-Gal-induced aging model. #, *p* < 0.01 for comparison of model group with control group. *, *p* < 0.01 for comparison of experimental group with model group.

ALT and AST, released from liver mitochondria and cytoplasm, are recognized as indicators that reflect liver damage and the degree of hepatocyte damage [20]. The activities of serum ALT and AST in mice in the model group were significantly higher than those of the control group (Figure 2). This result indicates the occurrence of liver damage after being fed D-Gal for 8 weeks. After the administration of osthole, the activities of ALT and AST were significantly reduced (*p* < 0.01), and the levels between the high-dose group and the VE group had no obvious differences. This result suggests that osthole can normalize ALT and AST activities in the serum of mice with D-Gal-induced liver injuries.

### 2.4. Effect of Osthole on Oxidative Stress in Liver Tissue 

**Figure 3:**
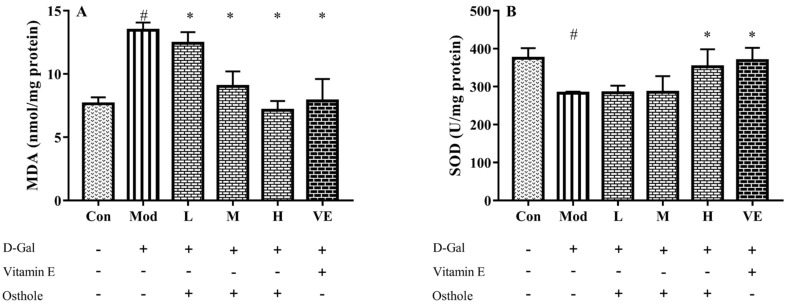

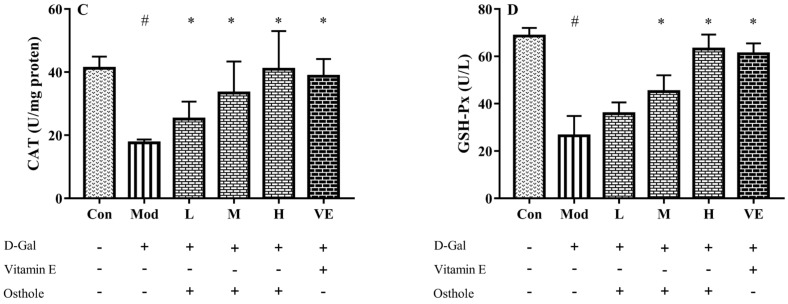
Effects of osthole on levels or activity of (**A**) MDA, (**B**) SOD, (**C**) CAT, and (**D**) GSH-Px in liver of mouse D-Gal-induced aging model. #, *p* < 0.01 for comparison of model group with control group. *, *p* < 0.01 for comparison of experimental group with model group.

In the aging mouse model, the excessive administration of D-Gal causes the accumulation of ROS, which leads to oxidative stress [21]. Accumulated oxidative stress leads to lipid peroxidation and destroys cellular components, which finally causes cell death [22]. Liver healthy status can be measured with the content of various enzymes and biochemicals related to oxidative stress in liver tissue. MDA is the biomarker of oxidative stress and reflects the degree of lipid peroxidation [23]. CAT can catalyze the decomposition of hydrogen peroxide into oxygen and H_2_O [24]. Meanwhile, GSH-Px is involved in the detoxification of metabolites produced from liver oxidative injury [25]. In this study, biomarkers reflecting degree of oxidation, such as superoxide dismutase (SOD), malondialdehyde (MDA), catalase (CAT), and GSH peroxide (GSH-Px), were measured. The administration of D-Gal significantly increased the levels of MDA, and reduced the activities of SOD, CAT and GSH-Px (Figure 3). Osthole restored the activity of the antioxidant enzymes in a dose-dependent manner, and the high-dose group (400 mg osthole/kg/BW) showed a significant increase in SOD and CAT activities compared to the model group. Meanwhile, osthole also decreased the MAD content in the liver tissue. Therefore, osthole effectively protects hepatocytes against D-Gal-induced liver injury.

### 2.5. Effect of Osthole on Pro-Inflammatory Cytokines in Serum and Liver Tissue

**Figure 4:**
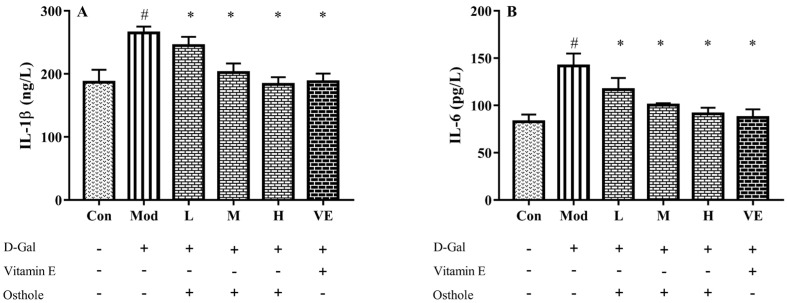
Effects of osthole on levels of (**A**) IL-1β and (**B**) IL-6 in serum of mouse D-Gal-induced aging model. #, *p* < 0.01 for comparison of model group with control group. *, *p* < 0.01 for comparison of experimental group with model group.

Some pro-inflammatory cytokines, such as TNF-α and IL-6, can act as autocrine feedback signals and enhance the immune response. In the immune system, IL-1β and IL-6 regulate the cross-talk of various inflammatory pathways as regulatory mediators [26]. According to previous studies, inflammation is always accompanied with organ injury, including lung [11] and renal injury [12]. In acute alcoholic liver injury, the increase in pro-inflammatory cytokine levels was also determined [27]. Similar to these studies, serum IL-1β and IL-6 levels of the model group were also significantly higher than those of the control group (Figure 4). Compared to the model group, osthole significantly reduced the levels of IL-1β and IL-6 in serum (*p* < 0.01). Meanwhile, the high dose of osthole showed no obvious difference between the control and VE group, indicating the need for a certain osthole dose.

**Figure 5:**
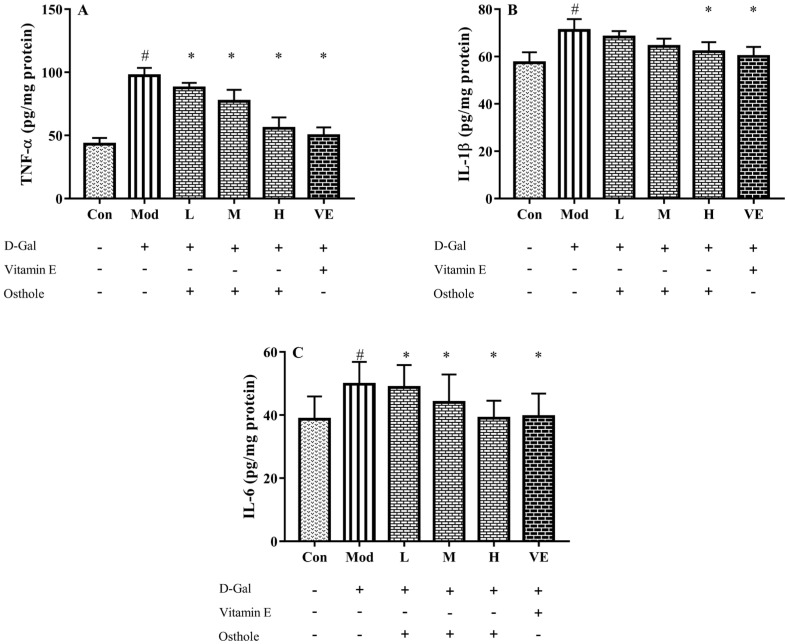
Effects of osthole on levels of (**A**) TNF-α, (**B**) IL-1β and (**C**) IL-6 in liver tissue of mouse D-Gal-induced aging model. #, *p* < 0.01 for comparison of model group with control group. *, *p* < 0.01 for comparison of experimental group with model group.

In the liver injury model, pro-inflammatory cytokines, such as TNF-α, IL-1β and IL-6, are abnormally expressed in liver tissue [15]. TNF-α plays an important role in the liver injury process by stimulating the production of other pro-inflammatory cytokines and leading to the apoptosis of hepatocytes [28]. After analyzing the levels of pro-inflammatory cytokines in serum, the effects of osthole on the expression of those in the liver tissue are noticed. In Figure 5, the levels of TNF-α, IL-1β and IL-6 in the liver tissue of the model group significantly (*p* < 0.01) increased compared to the control group. This result indicated a severe inflammatory reaction. After the osthole treatment, the results exhibited a significant decrease in detected pro-inflammatory cytokines compared to the model group (*p* < 0.01). Therefore, the results from the serum and liver tissue analysis suggest that osthole can alleviate inflammatory response and hepatocyte injury by reducing pro-inflammatory cytokine levels *in vivo*.

### 2.6. Effect of Oshtole on Signal Pathway-Mediated Inflammatory Response

**Figure 6:**
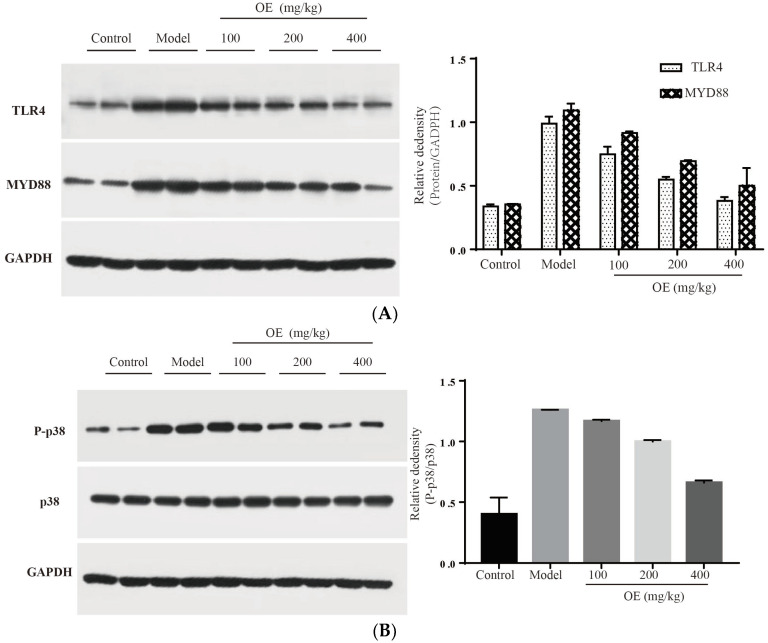

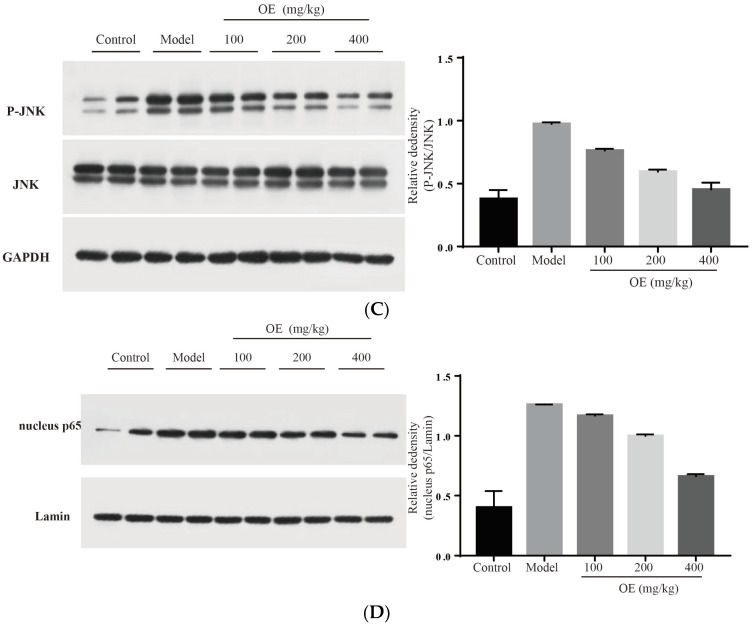
Effect of osthole on TLR4, NF-κB and MAPK signaling pathways. (**A**) TLR4 and MYD88, (**B**) P-p38, (**C**) p-JNK and (**D**) nucleus p65. All data are presented as the mean ± SEM.

In this study, we confirmed that D-Gal-induced liver injury *in vivo* is associated with inflammation, and especially the excessive expression of pro-inflammatory cytokines (TNF-α, IL-1β and IL-6). Therefore, signaling pathways related to the expression of these cytokines may be targets for osthole. To elucidate the molecular mechanism of osthole against D-Gal-induced liver injury, TLR4 and related downstream proteins were determined. The results indicated that osthole effectively down-regulates the expression of TLR4 and its downstream protein factor MYD88 (*p* < 0.01) (Figure 6A). After binding to MYD88, TLR4 triggers the activation of the downstream tumor necrosis factor receptor-related factor (TRAF6), resulting in the activation of two signal transduction pathways, e.g., MAPK and NF-κB [29,30]. As summarized by Sun et al. [5], mitogen-activated protein kinase (MAPK), and nuclear factor KB (NF-κB) are pivotal signaling pathways in inflammatory response. Therefore, the effects of osthole on the MAPK and NF-κB signaling pathways were investigated.

MAPK family proteins include p38, ERK and JNK, and play an essential role in liver injury, oxidative and apoptosis. In the model group, phosphorylation levels of p38 (P-p38) and JNK (P-JNK) were increased (Figure 6B,C), led to mitochondrial dysfunction, and finally induced hepatocyte apoptosis in the liver tissue [31]. After the administration of osthole, P-p38 and P-JNK were significantly down-regulated (*p* < 0.01). NF-κB is an important transcription factor in the inflammation process and regulates the expression of various cytokines and growth factors [32]. After activation by binding with phosphorylated IκB, NF-κB dimers (p65 and p50) translocate into the nucleus and then bind to the target location to initiate the transcription of pro-inflammatory cytokines. Compared to the control group, the levels of nucleus p65 significantly increased in the model group (*p* < 0.01), indicating the enhanced transcription and expression of pro-inflammatory cytokines (Figure 6D). After osthole intervention, the nucleus p65 level was effectively down-regulated, suggesting the suppressing role of osthole on the NF-κB signaling pathway. These results illustrated that MAPK, the NF-κB signaling pathway and the upstream proteins TLR4 and MYD88 are involved in exerting the protective effects of osthole in D-Gal-induced liver injury.

## 3. Materials and Methods

### 3.1. Materials

Osthole was purchased from Shanghai yuanye Bio-Technology Co. (Shanghai, China). Corn oil was obtained from Golden Dragon Fish (Taizhou, China). Commercial kits for serum biochemical index of alanine aminotransferase (ALT) and aspartate aminotransferase (AST) were purchased from Nanjing Jiancheng Technology Co. (Nanjing, China). ELISA kits for cytokines of IL-1β and IL-6 in serum and liver tissue were from Nanjing Jiancheng Technology Co. Commercial kits for superoxide dismutase (SOD), catalase (CAT) and malondialdehyde (MDA) were obtained from Nanjing Jiancheng Technology Co. Antibodies used in this study were purchased from Cell Signaling Technology (Beverly, MA, USA).

### 3.2. Animal Experiment

In this study, 48 ICR male mice were purchased from Hanzhou Ziyuan Biotech. Co. (Hanzhou, China) (SPF, SYXK (ZHE) 2018-0013). The mice were housed under standard SPF conditions, and then divided into the control (0.9% saline + corn oil), model (D-gal in 0.9% saline + corn oil), low-dose (D-gal in 0.9% saline + 100 mg/kg/BW osthole in corn oil), medium-dose (D-gal in 0.9% saline + 200 mg/kg/BW osthole in corn oil), high-dose (D-gal in 0.9% saline + 400 mg/kg/BW osthole in corn oil), and VE groups (D-gal in 0.9% saline + 150 mg/kg/BW vitamin E in corn oil). Before being fed osthole, the mice in all groups except the control were intraperitoneally administered 200 mg/kg/BW D-galactose once a day for 8 weeks to establish the D-Gal-induced liver injury model. After sacrificing, liver and blood samples were collected for subsequent experiments. The whole experiments were carried out according to the Guide for the Care and Use of Laboratory Animals (The Ministry of Science and Technology of China, 2006), and the proposal of this study was approved by the Committee of Takzhou University (No. TZXY-2021-20211007).

### 3.3. Histological Assessment

The collected liver tissues were fixed in 4% formaldehyde for 48 h and embedded in paraffin. The sliced 5 μm sections were stained with hematoxylin and eosin (HE), and observed under a light microscope (Leica Biosystems Co., China, Shanghai) to assess the degree of liver damage. Histopathological changes in the organs were determined using a microscope at 400 magnifications.

### 3.4. Biochemical Indexes Assay 

The collected blood samples were separated by centrifugation (2000× *g*, 4 °C for 15 min) and levels of ALT and AST were tested with respective kits. The analysis of IL-1β and IL-6 levels in serum was also carried out by the ELISA kit according to manufacturer’s instructions (Nanjing, China). 

The liver tissue was homogenized with 10 volumes of pre-chilled 1.15% potassium chloride solution, and the homogenates were centrifuged. The obtained supernatants were tested for MDA, SOD, CAT and GSH-Px content with the kits. The levels of IL-1β and IL-6 in liver tissue were also analyzed. All data were normalized to the total protein, and bovine serum albumin was used as standard.

### 3.5. Western Blot Analysis

The collected liver tissues of various groups were mixed with RIPA buffer containing a protease inhibitor cocktail to make a homogenate. The formed solution was centrifuged at 12,000× *g* for 20 min, and total protein contents were determined using the total protein assay kit (Nanjing Jiancheng Bioengineering Institute, Nanjing, China). Equal amounts (50 μg) of total protein were separated by 10% sodium dodecyl sulfate-polyacrylamide gel electrophoresis (SDS-PAGE) and then transferred to polyvinylidene difluoride (PVDF) membranes. The membranes were blocked by 5% skim milk, and sequentially treated with primary antibodies overnight at 4 °C. The membranes were washed with TBST buffer and incubated with horseradish peroxidase (HRP)-conjugated secondary antibodies for 1 h at room temperature. The positive bands were detected with an enhanced chemiluminescence solution.

### 3.6. Statistical Analysis

Data are expressed as mean ± SD. Comparisons between two groups were made by one-way analysis using GraphPad Prism software program V. 7.0. Differences with *p* < 0.01 were considered significant.

## 4. Conclusions

In summary, osthole exerts a protective effect against D-Gal-induced liver injury *in vivo*. Osthole treatment ameliorated oxidative stress by mediating levels or activities of MDA, CAT, SOD and GSP-Px, and inhibited levels of pro-inflammatory cytokines, such as TNF-α, IL-1β and IL-6, in serum and liver tissue. The protective effect of osthole against D-Gal-induced liver injury could be attributed to the regulation of TLR4/MAPK/NF-κB pathways. Our findings provide the underlying mechanism of osthole against liver injury, suggesting the potential value of this coumarin derivative as a therapeutic agent for aging liver injury.

## Supplementary Materials

The following supporting information can be downloaded at: https://www.mdpi.com/article/10.3390/molecules28010443/s1, Figure S1: Effect of osthole on the body weight and liver indexes in D-Gal-injected mice after 8 weeks of feeding. #, *p* < 0.01 for comparison of model group with control group. *, *p* < 0.01 for comparison of experimental group with model group.
