# Supplementary material for: Osthole Alleviates D-Galactose-Induced Liver Injury In Vivo via the TLR4/MAPK/NF-κB Pathways
Source: Molecules. 2023 Jan 3;28(1):443. doi: 10.3390/molecules28010443 (PMC9824625; doi:10.3390/molecules28010443)

Figure S1. Effect of osthole on the body weight and liver indexes in D-Gal-injected mice after 8-week feeding. #, $p < 0.01$ for comparison of model group with control group. *, $p < 0.01$ for comparison of experimental group with model group.

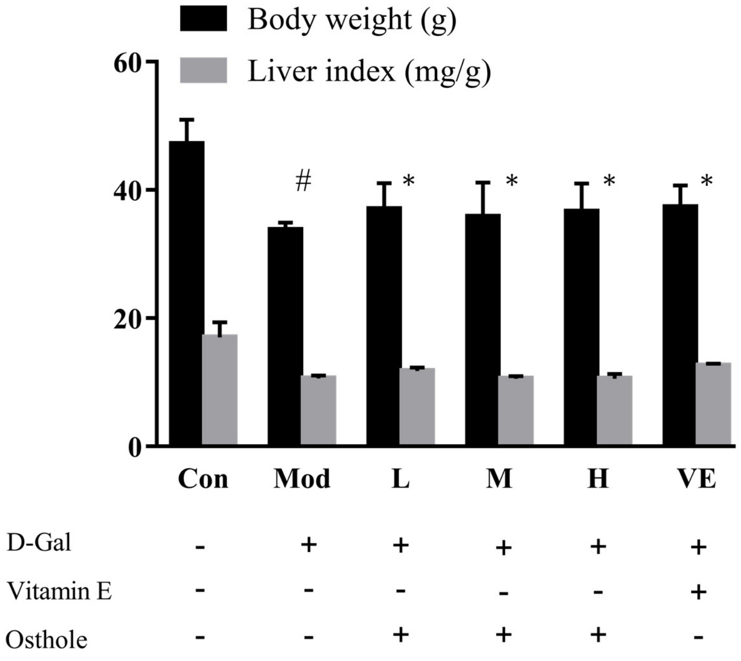

Supplement: Supplementary file 1 [file molecules-28-00443-s001.zip › molecules-2036863-supplementary.pdf]
